# Phenotypic Variability in iPSC-Induced Cardiomyocytes and Cardiac Fibroblasts Carrying Diverse *LMNA* Mutations

**DOI:** 10.3389/fphys.2021.778982

**Published:** 2021-12-16

**Authors:** Jiajia Yang, Mariana A. Argenziano, Mariana Burgos Angulo, Alexander Bertalovitz, Maliheh Najari Beidokhti, Thomas V. McDonald

**Affiliations:** ^1^Department of Molecular Pharmacology and Physiology, Morsani College of Medicine, University of South Florida, Tampa, FL, United States; ^2^Heart Institute, Department of Medicine (Division of Cardiovascular Sciences), Morsani College of Medicine, University of South Florida, Tampa, FL, United States

**Keywords:** *LMNA*, dilated cardiomyopathy, induced pluripotent stem cell, cardiomyocytes, cardiac fibroblasts, connexin 43

## Abstract

Mutations in the *LMNA* gene (encoding lamin A/C) are a significant cause of familial arrhythmogenic cardiomyopathy. Although the penetrance is high, there is considerable phenotypic variability in disease onset, rate of progression, arrhythmias, and severity of myopathy. To begin to address whether this variability stems from specific *LMNA* mutation sites and types, we generated seven patient-specific induced pluripotent stem cell (iPSC) lines with various *LMNA* mutations. IPSC-derived cardiomyocytes (iCMs) and cardiac fibroblasts (iCFs) were differentiated from each line for phenotypic analyses. *LMNA* expression and extracellular signal-regulated kinase pathway activation were perturbed to differing degrees in both iCMs and iCFs from the different lines. Enhanced apoptosis was observed in iCMs but not in iCFs. Markedly diverse irregularities of nuclear membrane morphology were present in iCFs but not iCMs, while iCMs demonstrated variable sarcomere disarray. Heterogenous electrophysiological aberrations assayed by calcium indicator imaging and multi-electrode array suggest differing substrates for arrhythmia that were accompanied by variable ion channel gene expression in the iCMs. Coculture studies suggest enhancement of the *LMNA* mutation effects on electrophysiological function exerted by iCFs. This study supports the utility of patient-specific iPSC experimental platform in the exploration of mechanistic and phenotypic heterogeneity of different mutations within a cardiac disease-associated gene. The addition of genetically defined coculture of cardiac-constituent non-myocytes further expands the capabilities of this approach.

## Introduction

Familial cardiomyopathy is the most common Mendelian inherited heart disorder. Among these, dilated cardiomyopathy (DCM) has been estimated between 1 in 250 to 2,500 of the general population (Hershberger et al., 2013). More than 50 genes have been associated with familial DCM encoding sarcomeric, cytoskeletal, nuclear and plasma membrane proteins (Mestroni et al., 2014; Orphanou et al., 2021). A prominent locus for familial DCM is *LMNA*, encoding lamin A and C (lamin A/C; McNally and Mestroni, 2017; Peters et al., 2019; Schultheiss et al., 2019). Lamin A/C is a type V intermediate filament protein and the major structural component of the nuclear lamina, that lies beneath the inner nuclear membrane (Fisher et al., 1986). Besides providing mechanical support to the nucleus, lamins play an essential role in chromatin organization, DNA repair, transcription, nucleo-cytoskeletal connections, and cellular signaling pathways (Dechat et al., 2008). Mutations in *LMNA* associate with a wide range of human diseases collectively referred to as “laminopathies” encompassing not only DCM, but also muscular dystrophy, lipodystrophy, peripheral neuropathy, and Hutchinson Gilford Progeria (premature aging syndrome; Capell and Collins, 2006; Rankin and Ellard, 2006).

*LMNA*-associated cardiomyopathy is progressive and highly penetrant (Hershberger and Morales, 1993). The end-stage disease is generally characterized by heart failure with severe systolic impairment, cardiac conduction defects, and tachyarrhythmias (atrial and ventricular; Kumar et al., 2016). Earlier stages of disease exhibit phenotypic variability in age of onset, rate of progression, and relative prominence of cardiac pathology. The first manifestation may be heart block, atrial fibrillation, congestive failure, or sudden death (Crasto et al., 2020). One potential factor in this phenotypic pleiotropy may be differing sites and types of mutations within *LMNA*. Clinical correlations suggest that different mutation sites and types within *LMNA* lead to variable cardiac phenotypes in humans (Nishiuchi et al., 2017 Captur et al., 2018). The clinical presentation of *LMNA*-associated DCM is often insidious, such that cardiac involvement may be well advanced upon first recognition. The first manifestation often carries considerable morbidity and mortality (Becane et al., 2000). Affected individuals are generally managed with standard heart failure medications (β-blockers, ACE inhibitors and diuretics) and antiarrhythmic strategies such as pacemakers or implanted cardiac defibrillators. Unfortunately, cardiac transplantation is often required for DCM patients caused by *LMNA* mutations with poor prognosis. Presently, there is no approved, specific optimal treatment for *LMNA* cardiomyopathy (Wang et al., 2017; Peters et al., 2019).

An initial insight into the pathophysiology of *LMNA* related DCM came from a transgenic mouse model (*Lmna*^H222P/H222P^) based on a missense mutation that was associated with Emery-Dreifuss muscular dystrophy in humans (Arimura et al., 2005). The *Lmna*^H222P/H222P^ mouse model exhibited phenotypes of human laminopathy including cardiac conduction defects, chamber dilation, and increased fibrosis. This model was further investigated and demonstrated extracellular signal-regulated kinase (ERK) hyperactivation and ERK inhibition delayed heart failure, improved survival in *Lmna*^H222P/H222P^ mice (Muchir et al., 2007, 2009, 2012a,b).

Much of the research to date, has been focused on *LMNA* function in cardiomyocytes. However, *LMNA* is expressed in a wide variety of cells. Furthermore, cardiac-resident, non-myocyte cells comprise about 50% of the cells of the heart and participate in normal cardiac function and exert influence on pathological states (Kamo et al., 2015). Thus, mechanistic studies to directly compare the effects of different *LMNA* mutations in cardiac myocytes and other cardiac-resident cells are merited to determine if alternate pathogenic pathways exist. To better understand specific genotype–phenotype differences in *LMNA*-DCM, we have recruited patients from affected families carrying a variety of *LMNA* mutations. We describe here our initial results from patient-specific induced pluripotent cells differentiated into cardiomyocytes (iCMs) and cardiac fibroblasts (iCFs) from 7 individuals. Our results support the concept that the molecular and functional pathways to heart disease vary with specific *LMNA* mutations. The diverse molecular, cellular, and electrophysiological signatures we observed may reflect mutation-specific differences in early molecular signaling, which merits further in-depth gene expression analyses with the aim towards more precise genotype-specific mechanism definition and potential therapeutic targets.

## Materials and Methods

### Reprogramming of PBMC and iPSC Culture

This study was reviewed and approved by the Institutional Review Board of the University of South Florida (IRB: Pro00033948) with participants’ written informed consent. We recruited seven subjects who were members of families affected by DCM associated with *LMNA* mutations. Detailed clinical information is provided in Table 1. Three control induced pluripotent stem cell (iPSC) lines were purchased from ATCC (ATCC-1026, ATCC-1028, ATCC-1029) without *LMNA* mutation.

**Table 1:** Clinical characteristics of subjects.

Subject AA change	Age	Sex	Mutation type	DCM	AF	VA	CCD	SMD	LGE	Presenting Symptom	Age onset
Control#1 ATCC 1026	31	M	Wild type	NA	NA	NA	NA	NA	NA	NA	NA
Control#2 ATCC 1028	31	F	Wild type	NA	NA	NA	NA	NA	NA	NA	NA
Control#3 ATCC 1029	24	F	Wild type	NA	NA	NA	NA	NA	NA	NA	NA
M1I	46	M	Altered start codon	+	+	+	+	+	+	AF/CHF	39 y
R216C /R399H	71	M	Missense	+	+	+	+	−	NA	Syncope/VT	62 y
R216C.m	45	M	Missense	−	−	−	+	−	−	None	Pre-clinical
R216C.f	34	F	Missense	−	−	−	−	−	−	Palpitations	Pre-clinical
R335Q	49	M	Missense	+	−	−	+	−	+	CHF	49 y
R377H	54	F	Missense	+	+	−	+	−	NA	Syncope/CCD	40 y
R541C	53	F	Missense	+	−	−	+	+	NA	CHF	15 y

Symbol (−) indicates absent; (+) present; DCM indicates dilated cardiomyopathy; CHF congestive heart failure; AF atrial fibrillation or flutter; VA ventricular arrhythmia; CCD cardiac conduction disease (AV block or bundle branch block); SMD skeletal muscle disease; NA not applicable or not available; AA amino acid; LGE late gadolinium enhancement on cardiac magnetic resonance imaging.

Peripheral blood mononuclear cells (PBMCs) were reprogrammed as previously described (Angulo et al., 2021; Argenziano et al., 2021; Yang et al., 2021a, b). Briefly, PBMCs were isolated from whole blood and transduced by Integration-free CytoTune^®^-iPSC Sendai Reprogramming Kit (Thermo Fisher) following the manufacturer’s protocol. Colonies were purified by handpicking. iPSCs were cultured on Matrigel (Corning) coated 6- or 12-well plates in mTeSR plus medium (STEMCELL Technologies) and were dissociated with DPBS containing 0.5 mm EDTA solution (Invitrogen). Cells were passaged every 3–5 days with ROCK inhibitor Y27632 (10 μm; Sigma-Aldrich). iPSCs used for differentiation ranged from Passage 20 to Passage 40. Cells were maintained at 37°C, 95% air, 5% CO_2_. Mycoplasma tests were routinely performed.

### Generation of Cardiomyocytes

Patient-derived and control iPSC lines were differentiated into iCMs using STEMdiff^™^ Cardiomyocyte Differentiation Kit (STEMCELL Technologies), as per the manufacturer’s protocol. Briefly, iPSCs were seeded on matrigel-coated 12-well plates and reached >95% confluency before starting differentiation. Cells were then treated with STEMdiff^™^ Cardiomyocyte Differentiation Medium A containing matrigel (1:100) for 2 days, STEMdiff^™^ Cardiomyocyte Differentiation Medium B for 2 days, STEMdiff^™^ Cardiomyocyte Differentiation Medium C for 4 days, and STEMdiff^™^ Cardiomyocyte Maintenance Medium. After 15 days of differentiation, cardiomyocytes were maintained in RPMI 1640 medium (Thermo Fisher) supplemented with B27 (Thermo Fisher) until day 30. Purification of cardiomyocytes was achieved by a metabolic-selection method as previously described (Sharma et al., 2015) with glucose starvation (RPMI-glucose + B27) for 5 days before functionality analysis. Fresh media was renewed every other day. Cardiomyocyte aggregates were dissociated using cardiomyocyte dissociation media (STEMCELL Technologies) for downstream experiments.

### Generation of Cardiac Fibroblasts

Cardiac fibroblasts were generated from iPSCs using the protocol described by the Wu group (Zhang et al., 2019). Briefly, iPSCs were seeded on Matrigel-coated 6-well plates and were cultured for 3–5 days until they reached 50–70% confluence when differentiation started (day 0). iPSCs were treated with 6 μm of CHIR99021 for 2 days, recovered in RPMI + B27-insulin for 24 h, and then treated with 5 μm of IWR1 (I0161, Sigma) for 2 days. On day 5, human induced pluripotent stem cell-derived cardiac progenitor cells (iPSC-CPCs) were switched to advanced DMEM medium (12,634,028, Gibco^®^, Life Technologies) supplemented with 5 μm of CHIR99021 and 2 μm of retinoic acid (R2625, Sigma-Aldrich) for 3 days, and recovered in advanced DMEM for 4 days. Human iPSC-EPCs were treated with 10 μm of FGF2 (100-18B, PeproTech) and 10 μm of SB431542 (S1067, Selleck chemicals) in a fibroblast growth medium (116–500, Cell applications) for another 6 days. Cells can be maintained in the fibroblast growth medium and passed 4–5 times for subsequent studies.

### Alkaline Phosphatase Staining and Immunofluorescence Analysis

For Alkaline Phosphatase staining, the iPSC colonies were fixed in 4% paraformaldehyde (w/v; Sigma-Aldrich) for 10 min and stained using Stemgent^®^ Alkaline Phosphatase Staining Kit II (REPROCELL). 50,000 cells/well were plated onto a 4-well chamber slide (NEST Scientific) and fixed with 4% paraformaldehyde for 10 min, permeabilized with 0.2% Triton X-100 (Sigma-Aldrich) for 10 min, followed by blocking in 1% BSA (Sigma-Aldrich), 22.52 mg/ml glycine (Fisher Scientific) in PBST (PBS + 0.1% Tween 20) for 30 min. Cells were stained with the primary antibody in 1% BSA overnight at 4°C, incubated with secondary antibody (Thermo Fisher) for 1 h at room temperature in the dark and mounted with VECTASHIELD HardSet Antifade Mounting Medium with DAPI (VECTOR Laboratories). Both primary and secondary antibodies were listed in Supplementary Table S1. Images were acquired with BZ-X800 Microscope (Keyence) or FluoView FV1000 Confocal Microscope (Olympus). Nuclear circularity was quantified with ImageJ software using the equation circularity = 4π*area/perimeter^2. Sarcomere organization was analyzed using TT power analyses with TTorg plugin in ImageJ (Pasqualin et al., 2015).

### Immunoblotting

Cells were lysed using RIPA buffer (Thermo Fisher) complemented with protease and phosphatase inhibitors (Thermo Fisher). Lysates were sonicated in short bursts and centrifuged at 15,000 rpm for 15 min. Protein concentration was measured using Bradford assay (Thermo Fisher). 20–50 ug of total protein was loaded per sample and separated on a 4–15% SurePAGE gradient gel (GenScript). The proteins were transferred onto nitrocellulose membrane using Trans-Blot Turbo Transfer System (Bio-Rad). REVERT stain (Li-COR) was used to normalize for total protein loaded. Membranes were blocked with PBS Odyssey Blocking Buffer (Li-COR) for 1 h at room temperature and incubated overnight at 4°C with primary antibodies, followed by secondary antibodies for 1 h at room temperature. Both primary and secondary antibodies were listed in Supplementary Table S1. Images were obtained using an Odyssey FC or CLX imaging system.

### Real-Time Quantitative PCR and Sanger Sequencing

The cell lysate was homogenized using the QiaShredder (Qiagen). RNA was isolated using RNeasy Mini Kit (Qiagen). The on-column DNase treatment was performed to remove the genomic DNA. cDNA was converted using the SuperScript VILO cDNA synthesis kit (Thermo Fisher). qPCR reaction was performed using Applied Biosystems TaqMan Fast Advanced Master Mix (Thermo Fisher) and run on the Step One Plus system (Applied Biosystems). Target gene expression levels were normalized to GAPDH mRNA levels and presented as relative to control cell lines in triplicate. GraphPad Prism was used for statistical analysis. The following TaqMan Assay probes (Thermo Fisher) were used: *GAPDH* (Hs02786624_g1), *GJA1* (Hs00748445_s1), *SCN5A* (Hs00165693_m1), *KCNH2* (Hs04234270_g1), *KCNQ1* (Hs00165003_m1), *ATP2A2* (Hs00544877_m1), *CASQ2* (Hs00154286_m1). For Sanger sequencing, RNA of iPSCs was extracted and converted to cDNA, amplified with DreamTaq polymerase (Thermo Fisher) and purified by Monarch PCR & DNA cleanup kit (New England Biolabs). Samples were sent for Sanger sequencing (Genewiz).

### Micro Electrode Array Electrophysiology

Micro Electrode Array (MEA) 24-well plates (Axion Biosystems) were coated with 5 μl droplets of fibronectin (F1141, Sigma-Aldrich) at 50 μg/ml onto each electrode region and incubated for 1 h at 37°C, 5% CO_2_. iCMs after metabolic selection at day 35 were seeded at 100,000 viable cells per well. For coculture, 90,000 iCMs at day 35 and 10,000 iCFs at passage 2 were mixed and plated on MEA per well. The cells were then incubated at 37°C/5% CO_2_ for 2 weeks with media renewed every 2 days. Field potential and propagation were recorded for 10 min from spontaneously beating monolayers using a Maestro MEA system (Axion Biosystems) at 37°C. The field potential duration was corrected (FPDc) for beating rate according to Fridericia’s formula: FPDc = FPD/Beat period^1/3^ (Asakura et al., 2015). Data from each cell line were collected from 3 wells and repeated three times. Three biological controls from a total of 27 wells were combined. Statistical analysis was performed using GraphPad Prism.

### Calcium Imaging

Intracellular calcium handling was studied using single-wavelength fluorescent calcium dye Fluo-4 AM (F14217, Thermo Fisher) diluted in anhydrous DMSO (Thermo Fisher) and imaged with BZ-X800 Microscope (Keyence). iCMs at day 35 were dissociated into single CMs onto Matrigel-coated 12-well plates. Four to 7 days post-dissociation, iCMs were loaded with 5 μm Fluo-4 AM in calcium-free Tyrode’s solution (NaCl 134 mm, KCl 5.4 mm, MgCl_2_ 2 mm, HEPES 10 mm, Glucose 10 mm) for 15–20 min and washed with Tyrode’s solution for 15 min at 37° C before imaging in Tyrode’s solution containing 2 mm Ca^2+^. Calcium imaging was recorded for 1–2 min at each field of interest. The mean intensity of the calcium transients was analyzed by drawing a region of interest over the whole cells using ImageJ software. The background noise was subtracted before further processing. The Ca^2+^ levels are presented as values of ΔF/F0. Bradycardia is defined as a beating rate 2 standard deviations lower than the average of the controls.

### Wound Healing Assay

The migration of human iCFs was determined using *in vitro* scratch assay (Liang et al., 2007). Three biological iCFs were used as controls. Briefly, after confluent monolayers of iCFs were formed, cells were scratched using a pipette tip (20 μl) to create a consistent gap width. Cells were then washed in PBS to remove residual cells in the wounded area. Images were captured using a transmitted-light microscope in identical areas at 0 h, 5 h, 10 h and 24 h post-scratch. The cell-free areas at each time point were measured by ImageJ software using the MRI wound healing tool (Suarez-Arnedo et al., 2020).

### Statistical Analysis

All experiments were repeated at least two times and data were expressed as mean ± standard errors of the means (S.E.M.). Statistical comparisons were determined using unpaired two-tailed Student’s t-test between two groups. Multiple comparison correction analysis was performed using one-way ANOVA followed by Tukey’s *post-hoc* test. Statistical significance was defined as a value of *p* < 0.05.

## Results

### Generation of Patient-Specific iPSCs and Generation of iPSC-Derived Cardiomyocytes and Cardiac Fibroblasts

**Figure 1:**
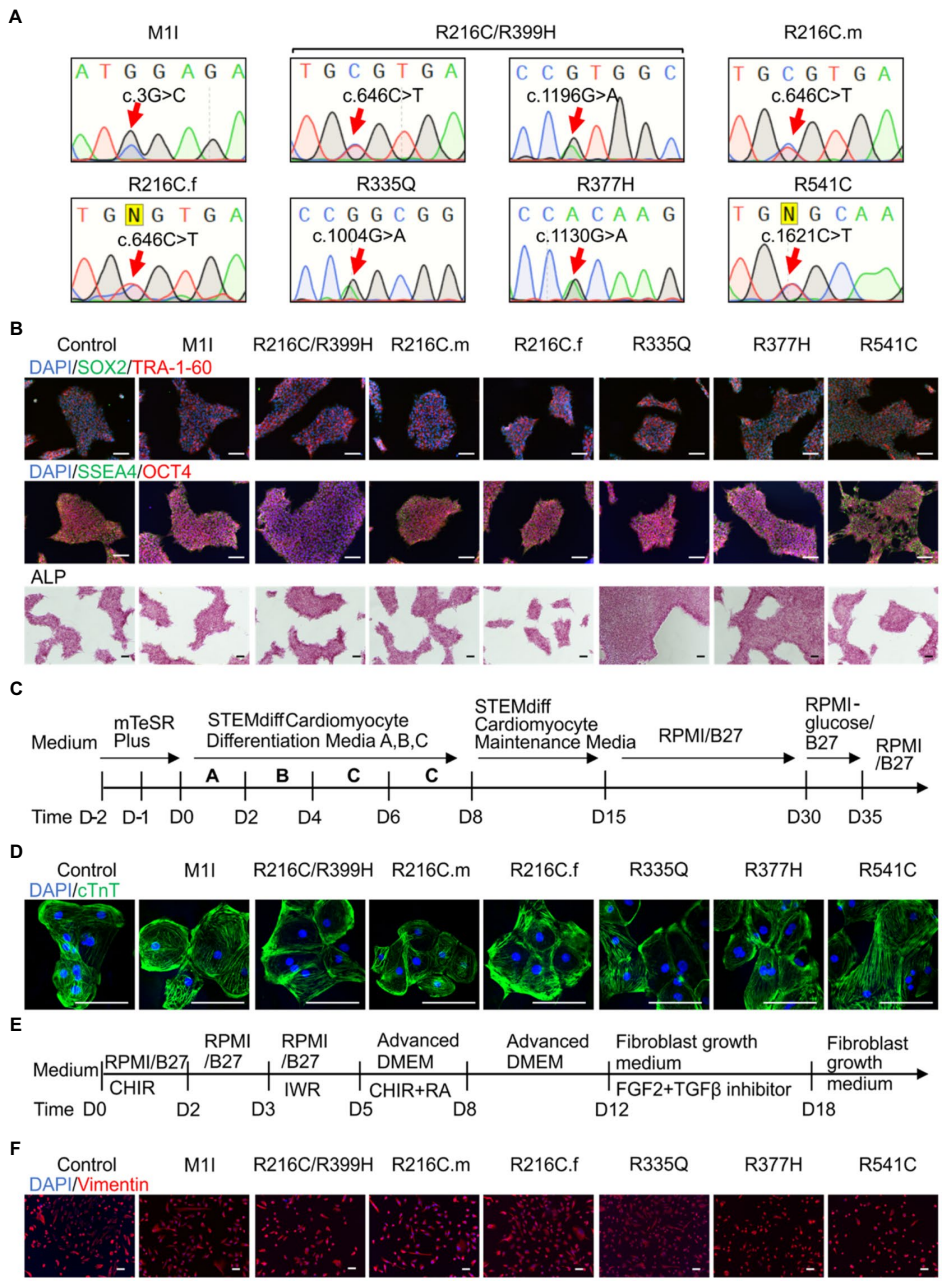
Differentiation and characterization of induced pluripotent stem cell (iPSC)-derived cardiomyocytes (iCMs) and cardiac fibroblasts (iCFs). **(A)** Sanger sequencing validates the presence of the individual mutation. **(B)** Representative staining of iPSCs expressing pluripotency markers SOX2 (green) and TRA-1-60 (red), SSEA4 (green), OCT4 (red), and alkaline phosphatase. Nuclei were stained with DAPI (blue). **(C)** Schematic of cardiac differentiation using STEMdiff^™^ Cardiomyocyte Differentiation kit. **(D)** Immunostaining of cardiac troponin T positive cardiomyocytes. **(E)** Workflow to induce cardiac fibroblasts using small molecule-based protocols. **(F)** Immunostaining of cardiac fibroblast specific marker, vimentin. Scale bar, 100 μm.

Blood samples obtained from seven subjects with DCM who were heterozygous for mutation in *LMNA* were used to generate iPSC clones. These missense point mutations in *LMNA* are M1I, R216C/R399H, R216C-male (R216C.m), R216C-female (R216C.f), R335Q, R377H and R541C. Table 1 illustrates demographic, and clinical information of the subjects. It is worthwhile noting that R216C.m and R216C.f are 2 unrelated subjects carrying the same mutation derived from different families. Genotyping by Sanger sequencing confirmed the presence of the specific mutation in *LMNA* at the expected site (Figure 1A). Three biological iPSC lines were used as controls and their absence of *LMNA* mutation was validated (data not shown). The verification of the pluripotent identity of R541C-iPSC has been previously published (Yang et al., 2021a). Likewise, all the cell lines showed positive staining of pluripotency markers including SOX2, TRA-1-60, SSEA4, OCT4 and alkaline phosphatase (Figure 1B). The characterization of one representative control line was shown. A normal karyotype was confirmed in all the mutant lines (Supplementary Figure S1A). PCR validated the presence of pluripotency markers including Oct4, Sox2, Klf4, Nanog and c-Myc (Supplementary Figure S1B). *In vitro* three germ-layer differentiation validated the iPSCs’ ability to differentiate into all three embryonic germ layers (Supplementary Figure S1C).

Patient-derived and control iPSC lines were differentiated towards iCMs using STEMdiff^™^ Cardiomyocyte Differentiation Kit, maintained in RPMI 1640 + B27 media until day 30 and selected by glucose starvation (Figure 1C). iCMs had spontaneous contractions and expressed the cardiac-specific marker troponin T by immunostaining (Figure 1D).

To establish cardiac fibroblasts, all iPSC lines were subjected to a small molecule-directed differentiation protocol (Zhang et al., 2019; Figure 1E). On day 18 of differentiation, the cells showed a typical fibroblast morphology. The presence of fibroblast-specific marker vimentin was confirmed by immunolabeling in all lines (Figure 1F).

### Different *LMNA* Cells Lines Exhibit Variable Levels of Lamin A/C, ERK Hyperactivation, and Cleaved Caspase-3 in iCMS and iCFs

**Figure 2:**
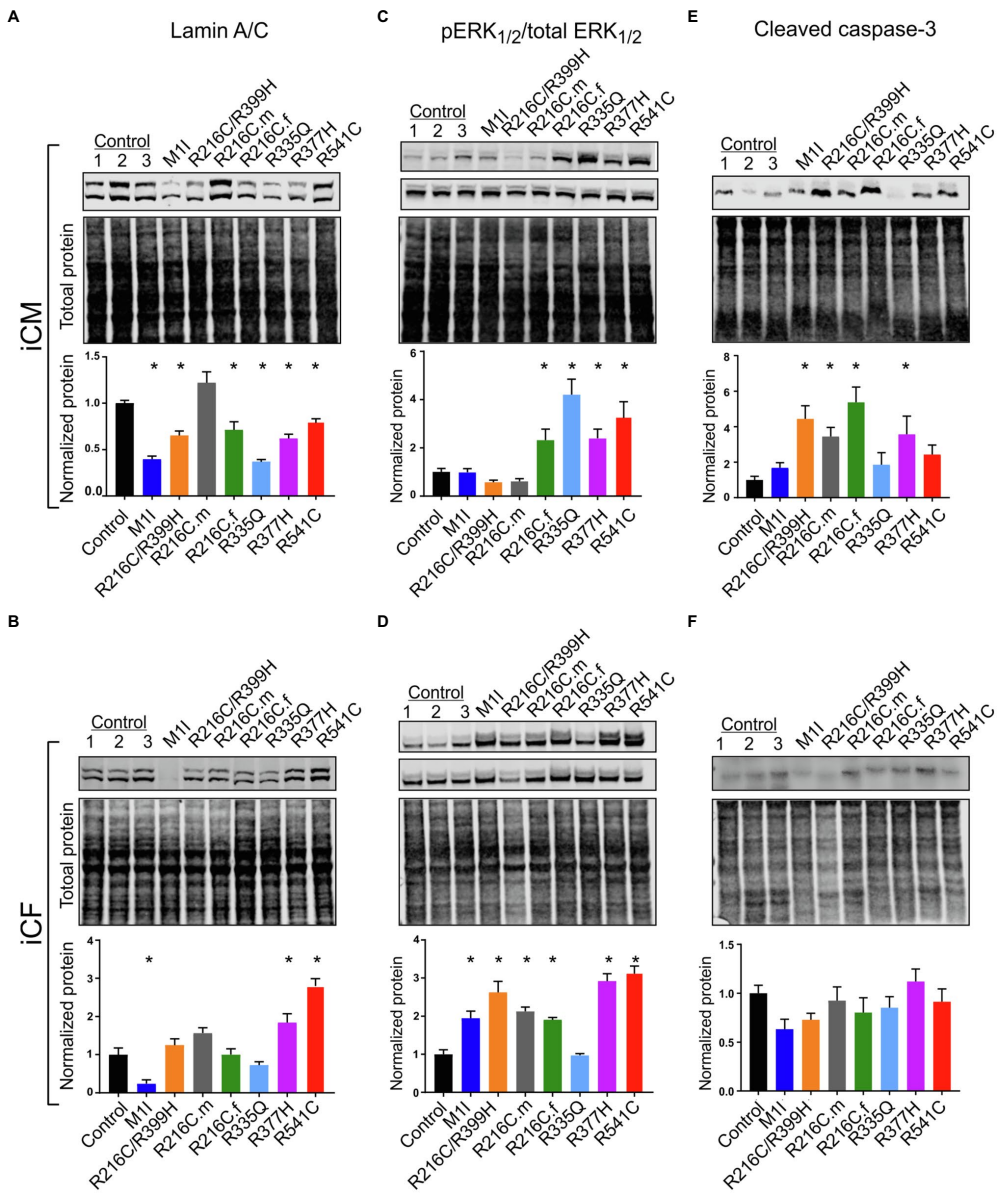
Variable levels of lamin A/C, extracellular signal-regulated kinase (ERK) pathway hyperactivation, and cleaved caspase-3 in different *LMNA*-iCMs and *LMNA*-iCFs. **(A)** Immunoblot showing lamin A/C and total protein expression in seven mutated and three control iCMs. The lower blot illustrates total protein loading. Histogram below blots demonstrates normalized lamin A/C expression quantification in three control iCMs and seven *LMNA* subjects. **(B)** Immunoblot of lamin A/C expression in iCF lines. Histogram below blots shows normalized lamin A/C expression quantification. **(C)** Immunoblot of phosphorylated ERK_1/2_ (pERK_1/2_ in top blot) and total ERK_1/2_ (in middle blot) from iCMs. Histogram below blots shows pERK_1/2_ activity in iCMs calculated as the ratio of pERK_1/2_ over total ERK_1/2_. **(D)** Immunoblot of pERK_1/2_ (top blot) and total ERK_1/2_ (middle blot) from iCFs. Histogram below blots shows pERK_1/2_ activation in iCFs calculated as in panel C. **(E)** Immunoblot for cleaved caspase-3 from iCMs (top blot). Histogram below blots shows quantitation normalized cleaved caspase-3 in iCMs. **(F)** Immunoblot for cleaved caspase-3 from iCFs as described for iCM in panel **(E)**. Combined results from three controls are shown. Results are expressed as means ± S.E.M. of three independent experiments. ^*^p < 0.05.

After we established iPSC-derived cardiomyocytes (iCMs) and cardiac fibroblasts carrying different *LMNA* mutations, we sought to compare their protein expression of lamin A/C. Three biological wildtype control cell lines were combined and averaged for analysis. Immunoblot indicated a significantly lower quantity of lamin A/C protein in all *LMNA* mutant iCMs except variant R216C.m compared to the wildtype iCMs (Figure 2A). Among all the variants of cardiac fibroblasts, lamin A/C expression was substantially declined in M1I but was significantly elevated in R377H and R541C relative to controls (Figure 2B).

ERK pathway hyperactivation has been implicated in the pathophysiological perturbation of cardiac laminopathies (Siu et al., 2012; Chatzifrangkeskou et al., 2018). We therefore evaluated ERK signaling cascade by calculating the ratio of phosphorylated ERK_1/2_ (pERK_1/2_) over total ERK_1/2_ through immunoblotting in iCMs and iCFs for all the subjects. In agreement with previous findings, the *LMNA*-iCMs had significant upregulation of pERK/ERK relative to controls in patients R216C.f, R335Q, R377H and R541C (Figure 2C). Markedly, the presence of ERK pathway hyperactivation was more prevalent in *LMNA*-mutant iCFs, with only R335Q showing no difference compared to controls (Figure 2D).

Increased apoptosis has been observed as a possible mechanism of *LMNA*-associated pathophysiology (Siu et al., 2012; Lee et al., 2017). Immunoblotting was performed to quantify the expression of cleaved caspase-3, a critical executioner of apoptosis, in iCMs and iCFs. In iCMs, patient R216C/R399H, R216C.m, R216C.f and R377H exhibited a higher degree of apoptotic marker compared to other *LMNA* variants or controls (Figure 2E). In iCFs, by contrast, there was no elevation in cleaved caspase-3 compared to control lines and there was no significant difference among the iCF cell lines (Figure 2F).

These results suggest that cardiac fibroblasts might be more significantly involved in the pathobiology of *LMNA*-related DCM concerning the abnormally activated ERK pathway than previously recognized. Increased levels of apoptosis markers in *LMNA*-iCMs are consistent with previous evidence. Further, this data supports that different *LMNA* mutation locations may not affect the same molecular pathways equally.

### Cellular Morphological Perturbations in *LMNA*-iCMs and iCFs

**Figure 3:**
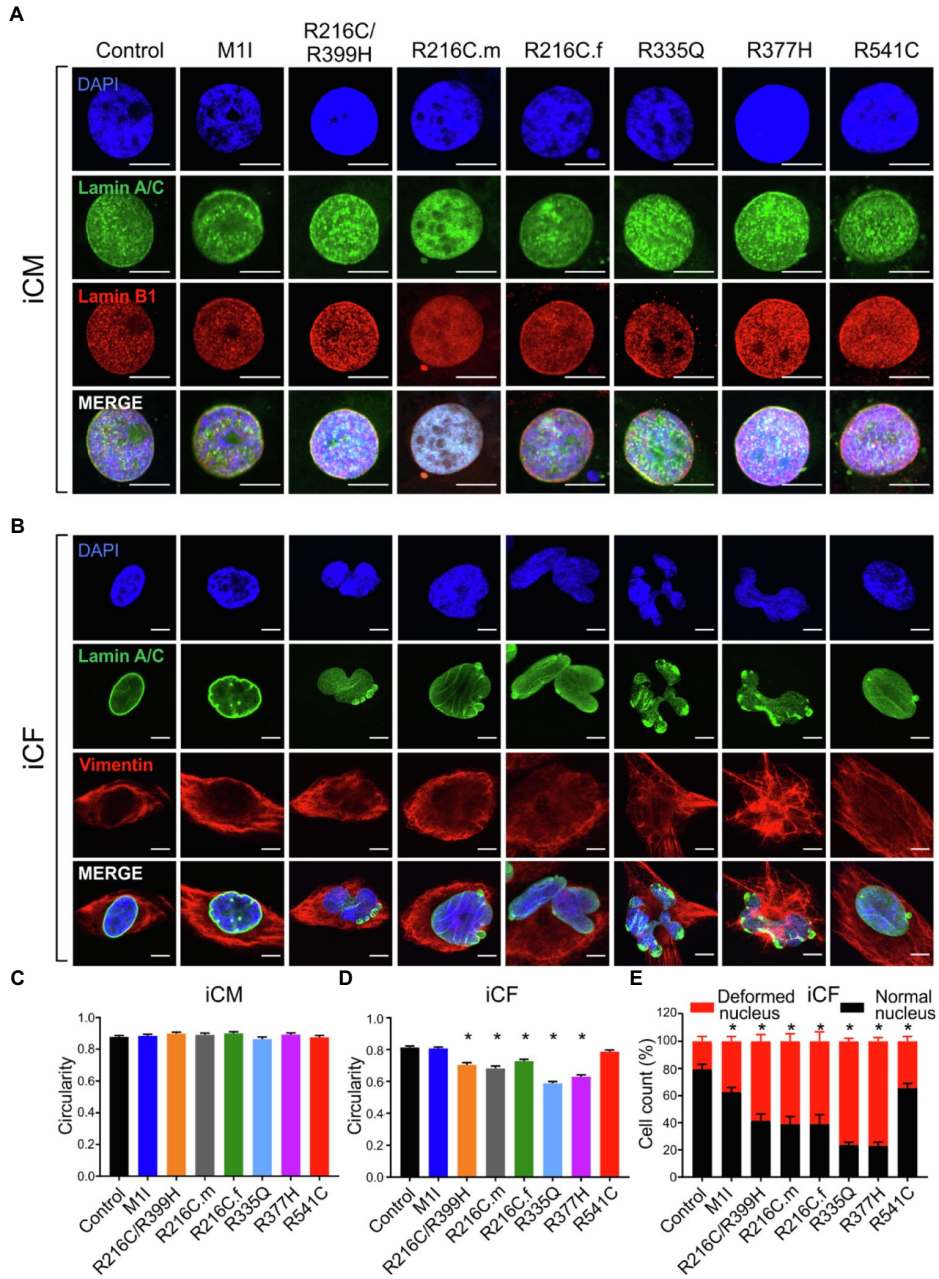
Characterization of lamin A/C nuclear structure in iCMs and iCFs. **(A)** Representative immunofluorescence micrographs of lamin A/C (green) and lamin B1 (red) in iCMs. Nuclei were stained with DAPI (blue). **(B)** Representative immunofluorescence micrographs of lamin A/C (green) and vimentin (red) in iCFs. Nuclei were stained with DAPI (blue). **(C)** Scoring of iCMs nuclear morphology assessed by circularity (*n* > 100). **(D)** Scoring of iCFs nuclear morphology assessed by circularity (*n* > 100). **(E)** Quantification of iCFs displaying abnormal nucleus. Combined results from three controls are shown. Data are expressed as means ± S.E.M. of two independent experiments. ^*^p < 0.05. Scale bar, 10 μm.

Previous studies on mature adult CMs and iPSC-derived CMs from patients affected with other *LMNA* mutations have shown aberrant nuclear morphology (Siu et al., 2012; Lee et al., 2017). We did not observe significantly misshapen nuclei in our *LMNA*-iCMs matured for 35–40 days as measured by fluorescence microscopy (Figures 3A,C). The *LMNA*-iCFs however, demonstrated irregular *LMNA* distribution and nuclear envelope shape in immunostaining for Lamin A/C antibody. Vimentin was co-stained to confirm the fibroblasts’ identity (Figure 3B). We observed an obvious wide range of nuclear dysmorphisms associated with different *LMNA* mutations in iCFs, such as nuclear blebbing, nuclear invagination, and atypical lamin A/C distribution. The nuclear deformity and lamin A/C aggregation at the peripheral nucleus were especially dramatic in variants R335Q and R377H. Objective assessment of reduced nuclear circularity supported the nuclear shape irregularities in variant R216C/R399H, R216C.m, R216C.f, R335Q and R377H (Figure 3D). Additionally, quantification of the diverse abnormal nucleus further confirmed an increased percentage of lamina structure impairment in all the *LMNA*-iCFs (Figure 3E).

**Figure 4:**
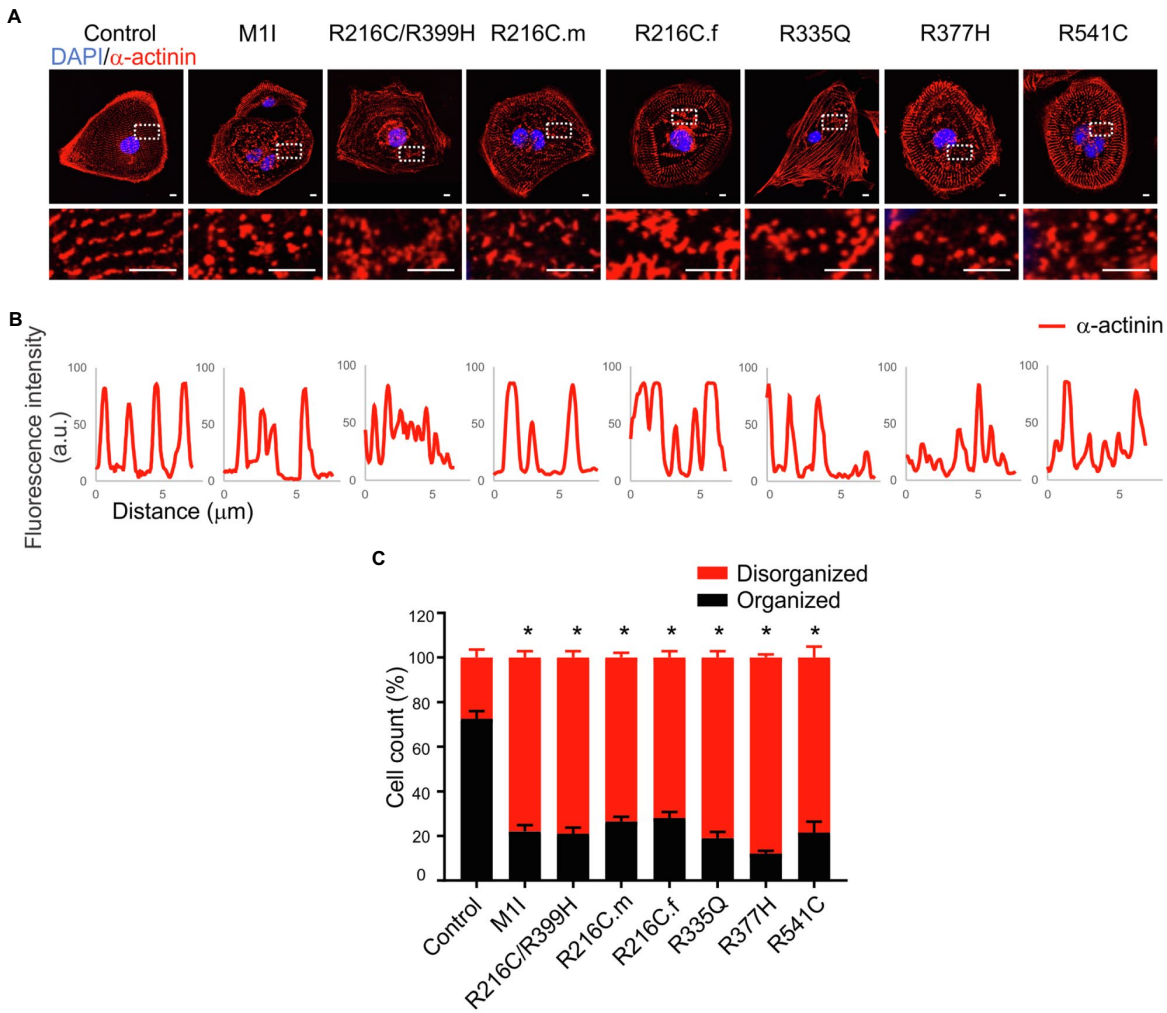
Immunofluorescence analysis of iPSC-CM sarcomere structure. **(A)** α-actinin (red) staining of cardiomyocytes revealed sarcomeric abnormalities (fragmented and punctuated staining) in *LMNA* mutant-iCMs. Nuclei were stained with DAPI (blue). Scale bar, 5 μm. Insets illustrate magnified areas in dotted white boxes (scale bar, 5 μm). **(B)** Line scans show striation patterns for sarcomeric α-actinin distributions (y-axis, the fluorescence intensity in arbitrary units; x-axis, distance in μm). **(C)** Immunostaining quantification shows the percentages of the disorganized sarcomere in mutant iCMs compared to controls (*n* > 20). Combined results from three controls are shown. Data are expressed as means ± S.E.M. of two independent experiments. ^*^p < 0.05.

Sarcomere architecture in iCMs was assessed by immunostaining using the α-actinin antibody (Figure 4A). Various *LMNA* mutations resulted in altered myofilament structures, suggesting irregular sarcomere organization (Figure 4B). The number of cells with severe sarcomeric abnormalities was significantly increased in all DCM iPSC-CMs compared to control cell lines (Figure 4C).

### Mutations in *LMNA* Reduce Migration Rate in iCFs

**Figure 5:**
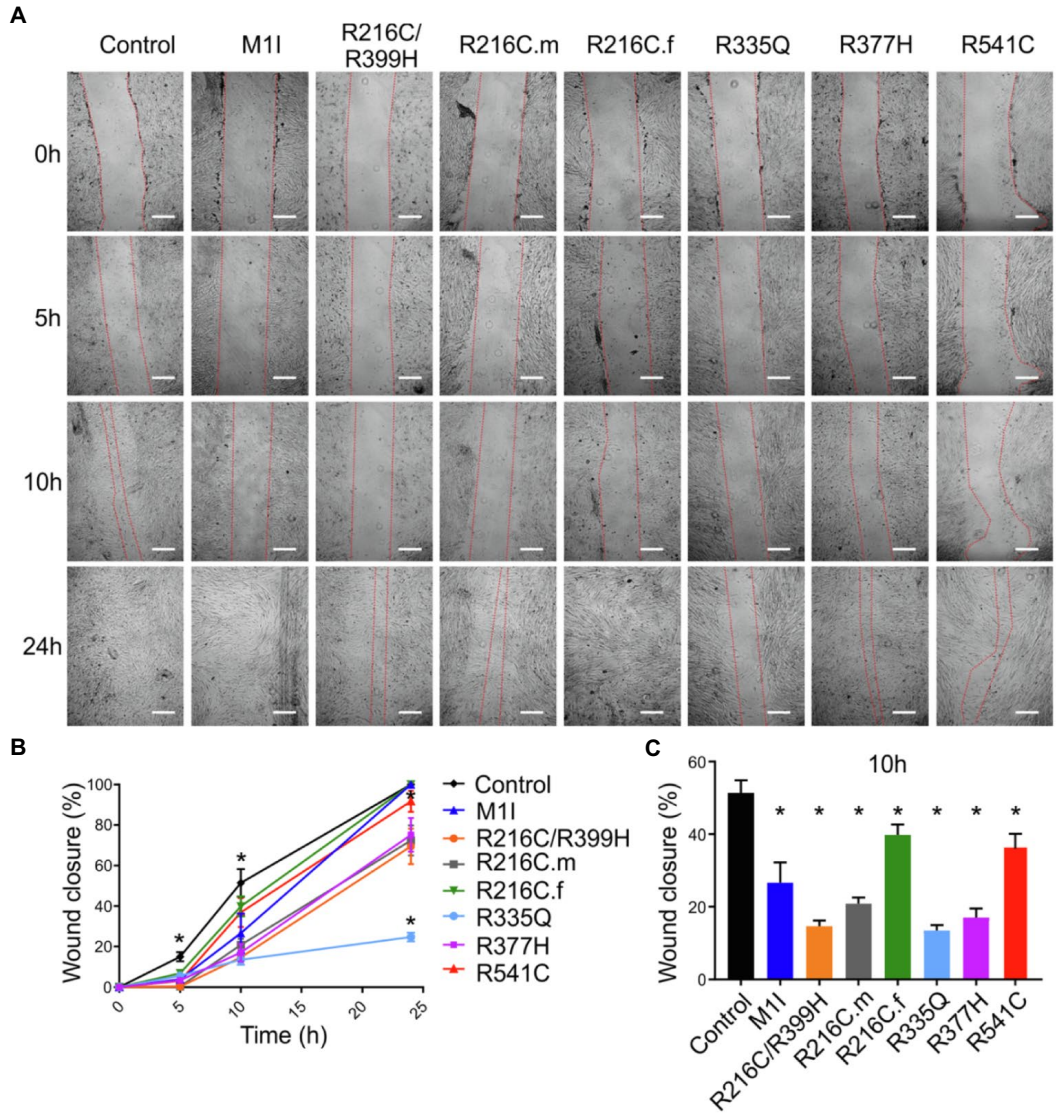
Reduced migration rate in *LMNA* mutated-iCFs. **(A)** Representative images showing diverse lines of iCFs migration at 0 h, 5 h, 10 h and 24 h. One representative control cell line was shown. Scale bar, 200 μm. **(B)** Quantitative data showing reduced migration rate in mutant-iCFs compared to control-iCFs over 24 h. **(C)** Histogram showing reduced wound closure area in mutant-iCFs compared to control-iCFs at 10 h. Combined results from three controls are shown. Data are expressed as means ± S.E.M. of three independent experiments. ^*^p < 0.05.

To assess functional abnormalities in iCFs, a wound-healing assay was conducted to measure cell migration rate (Figure 5A). Quantitative data showed a significant reduction in the migration rate over time in *LMNA*-iCFs (Figure 5B). This reduction was most prominently observed at the 10-h timepoint after the scratch (Figure 5C).

### Abnormal Calcium Handling in Individual Cells Reveals Arrhythmic Phenotype of *LMNA*-iCMs

**Figure 6:**
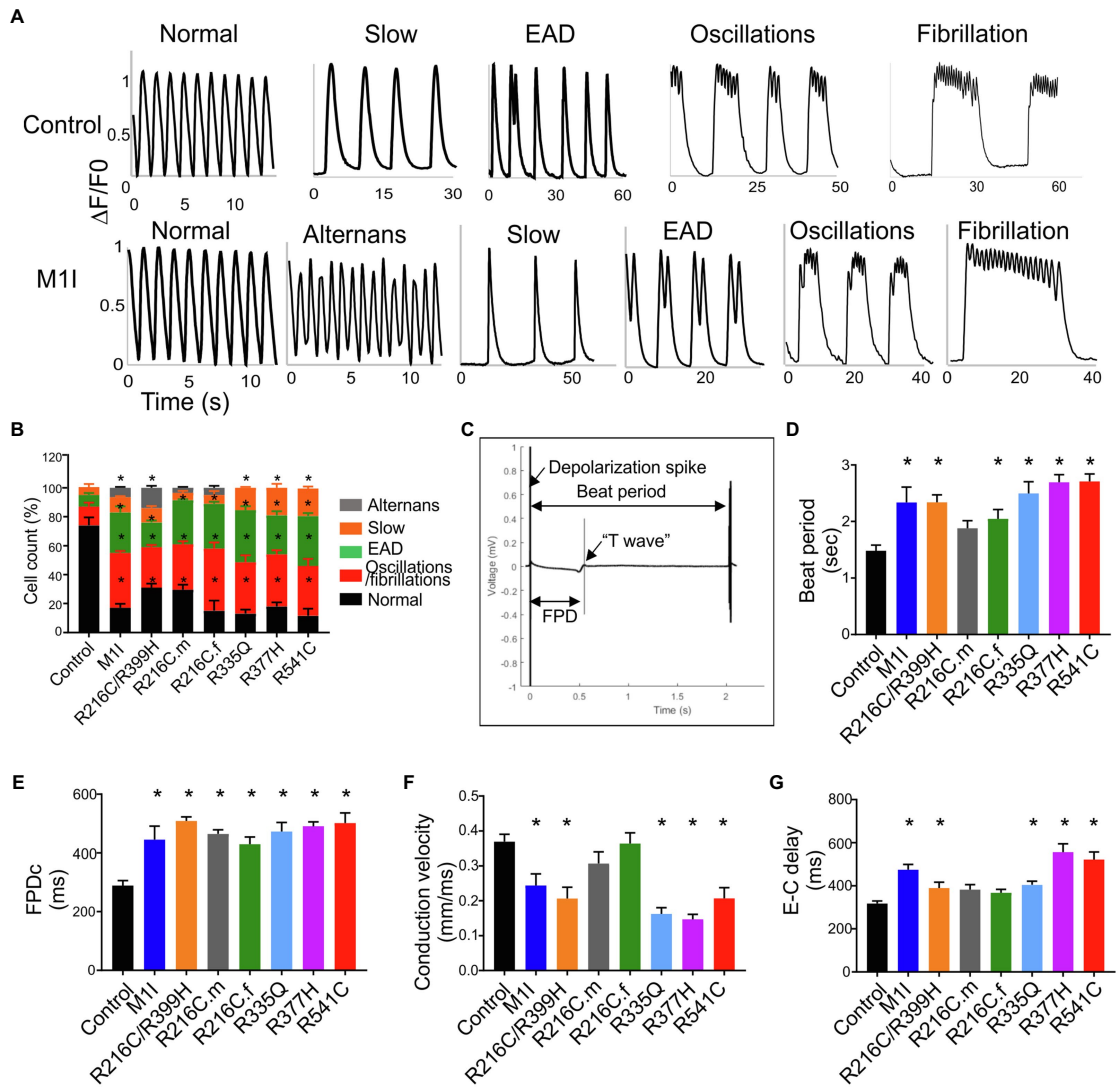
Analysis of calcium handling and electrophysiological characteristics on micro electrode array in all iPSC-CMs. **(A)** Representative calcium transient from *LMNA* mutant- and control-iCMs showing (from left to right) normal, alternans, slow, early afterdepolarizations, oscillations, and fibrillations. **(B)** Quantification of the frequency of various proarrhythmic events in all iCM lines (*n* > 40). **(C)** Representative extracellular field potential recording of the iPSC-CM monolayers on an MEA showing beat period, depolarization spike, repolarization bump (T-wave) and field potential duration. Histograms show the distribution of recorded **(D)** Beat period, **(E)** Field potential duration corrected (FPDc), **(F)** Conduction velocity, **(G)** Excitation-contraction delay (E-C delay); *n* = 3 wells per group. Combined results from Control #1, Control #2 and Control #3 are shown as control. Data are expressed as means ± S.E.M. of three independent experiments. ^*^p < 0.05.

We investigated the intracellular Ca^2+^-handling properties of the single dissociated iCMs by Fluo-4 calcium imaging. Besides regular Ca^2+^ transients (Figure 6A), *LMNA*-iCMs exhibited a wide variety of impaired calcium dynamics including alternans, slow beat rate, as well as more perturbed dysrhythmias such as early afterdepolarization, oscillations, and fibrillations (Figure 6A). Analysis of calcium transients demonstrated that *LMNA* mutants displayed a prominently higher frequency of these potentially proarrhythmic events (Figure 6B). Collectively, these findings indicate that dysregulation of intracellular calcium signalling exists at the cellular level and may provide an arrhythmogenic substrate for clinical disturbances in *LMNA*-DCM.

### The Mutations in *LMNA* Result in Electrophysiological and Ion Channel Alterations

The electrophysiological phenotype in *LMNA*-iCMs was investigated by the generation of field potentials at the multi-cellular level with MEA. A representative control tracing of extracellular field potential duration (FPD) was shown (Figure 6C). Based on the baseline electrophysiological characteristics, the *LMNA*-iCM aggregates displayed increased beat period (diminished beating rates) and prolonged FPD compared to the aggregates from controls (Figures 6D,E). As for conduction properties, iCMs from M1I, R216C/R399H, R335Q, R377H and R541C displayed reduced conduction velocity, as well as a prolonged excitation-contraction delay (Figures 6F,G), compared to healthy control iPSC-CMs.

**Figure 7:**
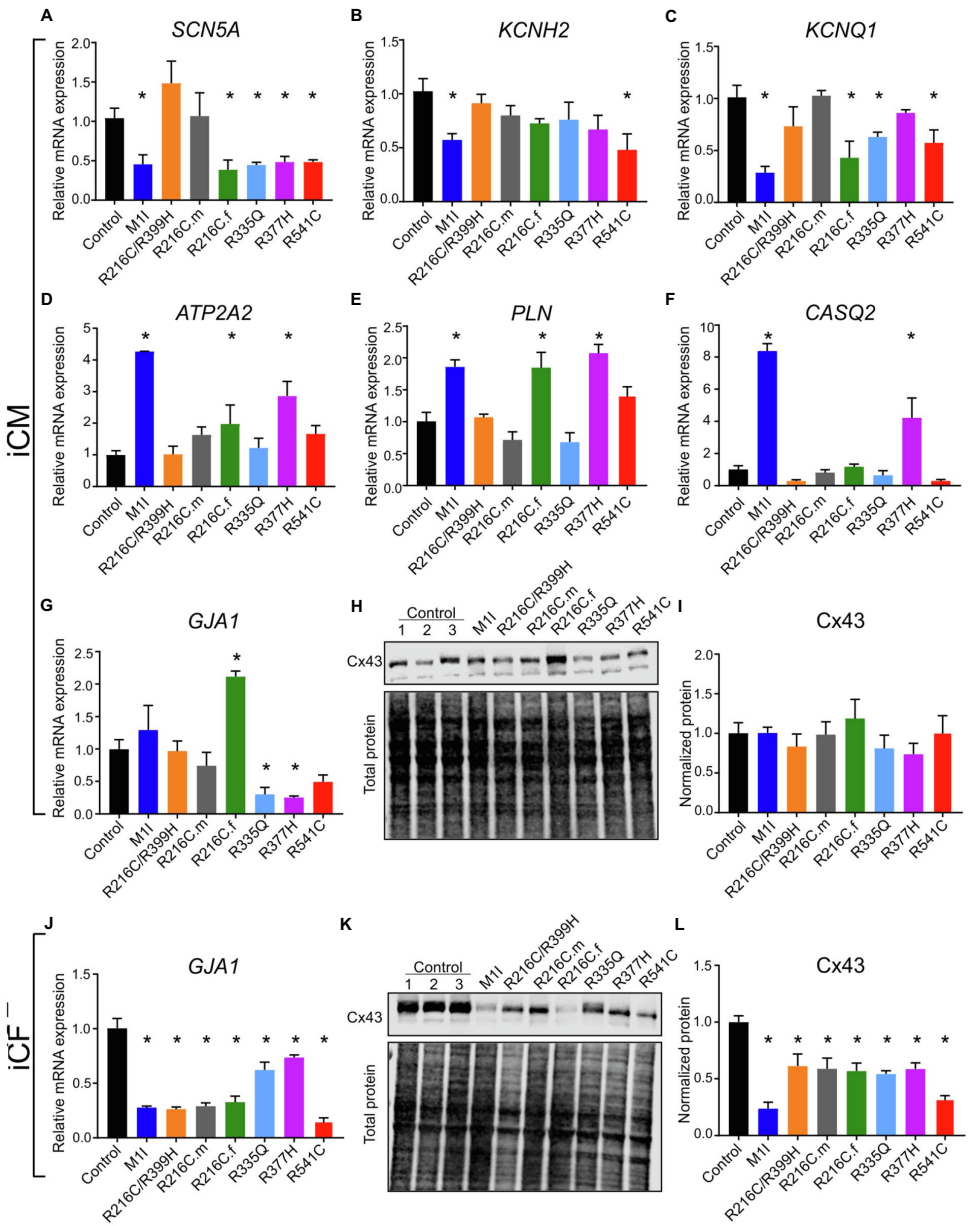
Analysis of ion channel levels. RT-PCR data showing mRNA transcripts in iCMs of **(A)** *SCN5A*, **(B)** *KCNH2*, **(C)** *KCNQ1*, **(D)** *ATP2A2*, **(E)** *PLN*, **(F)** *CASQ2*, **(G)** *GJA1*. **(H)** Immunoblot showing connexin 43 (Cx43) expression in seven mutated and three control iCMs. **(I)** Densitometry of Cx43 levels in all iCM lines normalized to total protein and relative to three combined controls. **(J)** *GJA1* mRNA quantification in different iCF lines by RT-PCR. **(K)** Immunoblot showing Cx43 expression in seven mutated and three control iCFs. **(L)** Densitometric analysis of Cx43 levels in all iCF lines. Combined results from Control #1, Control #2 and Control #3 are shown as control. Data are expressed as means ± S.E.M. of three independent experiments. ^*^p < 0.05.

Given the contributions of ion channel expression to electrophysiology characteristics, we examined the mRNA levels of several ion channels genes. *SCN5A*, the coding gene for fast voltage-dependent sodium channels (Nav1.5), exhibited variably reduced transcripts among different *LMNA* subject iCMs (Figure 7A). This finding is consistent with altered beating rates and conduction velocities. Meanwhile, the extension of corrected FPD in M1I and R541C may be attributed to depressed mRNA levels of *KCNH2* and *KCNQ1* (Figures 7B,C) two important potassium channels responsible for the repolarization phase of the cardiac action potential.

To study the genes involved in the regulation of the contraction/relaxation cycle, we evaluated the genes involved in calcium signalling including *ATP2A2*, *PLN* (phospholamban) and *CASQ2* (calsequestrin 2). *ATP2A2* encodes an enzyme called sarcoplasmic/endoplasmic reticulum calcium-ATPase 2 that mediates translocation of calcium from the cytosol into the sarcoplasmic reticulum lumen. Phospholamban is a key regulator of cardiac contractility and modulates sarcoplasmic Ca^2+^ sequestration. Calsequestrin is a calcium-binding protein that acts as a calcium buffer within the sarcoplasmic reticulum. Interestingly, *LMNA*-M1I and -R377H iCMs showed a significant elevation of mRNA levels of *ATP2A2*, *PLN* and *CASQ2* by qPCR (Figures 7D–F). These findings may imply the effect of calcium handling perturbations on electrophysiological properties.

Connexin 43 (Cx43), an important component of cardiac gap junctions, has been implicated in the pathogenesis of *LMNA* mutation (Sun et al., 2010; Chen et al., 2013; Macquart et al., 2019). *GJA1*, the gene encoding Cx43, exhibits reduced expression in iCMs from R335Q and R377H (Figure 7G). All mutant *LMNA* cell lines exhibit reduced GJA1 expression in iCFs relative to controls (Figure 7J). With regards to the expression of Cx43 protein, there were no significant differences observed in the mutant *LMNA*-iCMs in comparison to the control iCMs (Figures 7H,I). However, the protein abundance of Cx43 was markedly decreased in all the mutant *LMNA*-iCFs relative to the control iCFs (Figures 7K,L), suggesting that cell-cell communication in CFs may play an important role in *LMNA*-DCM pathophysiology.

### Coculture of iCFs and iCMs Carrying *LMNA* Mutations Show Exaggerated Electrical Disturbances

**Figure 8:**
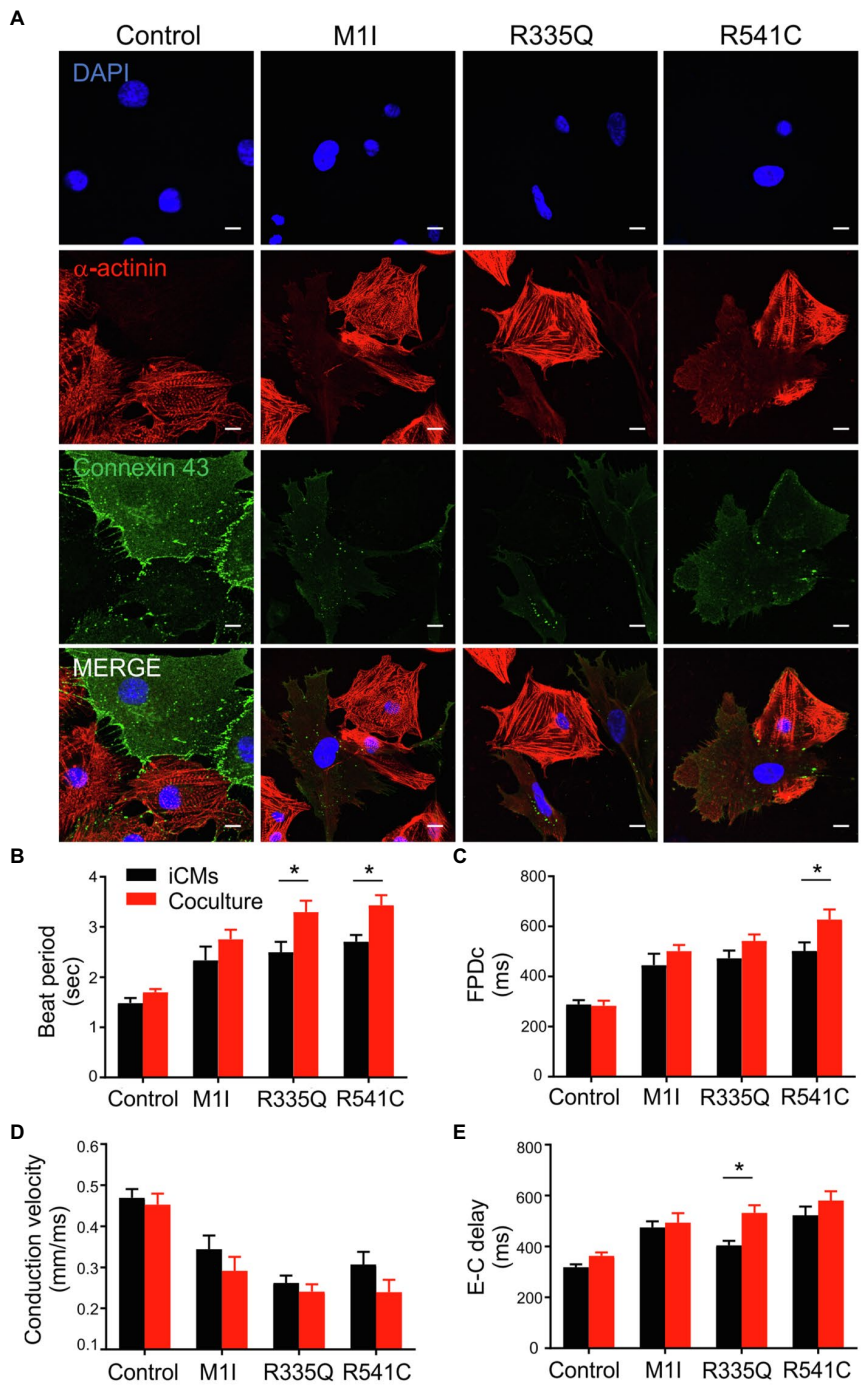
Analysis of coculture of iCMs and iCFs. **(A)** Representative immunofluorescence images of α-actinin (red), and connexin 43 (green) of iCMs and iCFs coculture from one representative control and three mutants M1I, R335Q, R541C. Nuclei stained with DAPI (blue). Scale bar, 10 μm. Bar graphs of **(B)** Beat Period, **(C)** FPDc, **(D)** Conduction velocity, **(E)** E-C delay (Excitation-contraction delay) of iCMs alone and coculture; *n* = 3 wells per group. Combined results from Control #1, Control #2 and Control #3 are shown as control. Data are expressed as means ± S.E.M. of three independent experiments. ^*^p < 0.05.

To examine the potential crosstalk between iCMs and iCFs, we performed coculture experiments by combining 90% iCMs and 10% iCFs from the identical parent iPSC lines. As described previously, immunoblotting/qPCR showed perturbed Cx43 expressions in *LMNA*-iCFs more prominently than in *LMNA*-iCMs. We, therefore, hypothesized that iCFs coupling to iCMs *via* Cx43-mediated gap junctions could contribute to the electrical disturbances caused by *LMNA* mutations. We focused on three mutations (M1I, R335Q and R541C) and three biological controls. Immunofluorescent co-staining illustrated cardiac-specific marker α-actinin and Cx43 was performed on the cocultured cells. The fluorescence signal of Cx43 was markedly weaker in the *LMNA* mutant patients than the wild-type controls (Figure 8A). Of note, although Cx43 was mainly localized at the cell-cell contacts in control coculture, it was more confined to the cytoplasm in *LMNA* mutant cells. Next, we further examined the electrophysiology characteristics of the cocultured cells on MEA. Interestingly, compared to the controls, M1I-, R335Q- and R541C-*LMNA* mixture monolayer showed a trend towards exaggerated electrical perturbations over iCMs alone, including prolonged beat period and FPDc, slower conduction velocity accompanied by increased excitation-contraction delay (Figures 8B–E). These results suggest that the *in vitro* electrical response of *LMNA* mutant cardiomyocytes was enhanced by *LMNA*-iCFs, possibly due to the lower abundance of Cx43.

## Discussion

Laminopathies involving multiple systems and organs present with extremely high penetrance and variable expressivity among different families. Cardiac defects eventually occur in a large proportion of symptomatic individuals (Lu et al., 2011). Among the unmet needs are gene-specific and variant-specific mechanisms, predictive biomarkers, and pre-emptive management strategies for pre-clinical gene carriers. Extensive research using the *Lmna*^H222P/H222P^ mouse model has elucidated pathogenic pathways leading to clinical phenotypes and other groups have reported models of *LMNA* cardiomyopathy, including the use of iPSC systems. Patient-specific iPSC-derived CM models of *LMNA*-DCM have shown nuclear blebbing and apoptosis (Siu et al., 2012), ERK_1/2_-dependent phosphorylation of cofilin (Chatzifrangkeskou et al., 2018), epigenetic suppression of *SCN5A* expression (Salvarani et al., 2019), and *PDGF*-dependent perturbation of calcium signaling (Lee et al., 2019). These studies each suggest a variety of mechanisms that could be mutation-specific, however, no side-by-side comparison of different *LMNA* mutations has been evaluated under the same conditions to accurately address this question.

In this study, we compare the molecular, cellular, and physiological phenotypes in iPSC models from seven different carriers of *LMNA* mutations side by side. Cardiac fibroblasts are a prominent non-myocyte cell type in the heart estimated to range from 20–60% (Pinto et al., 2016; Zhou and Pu, 2016). Massive cardiac fibrosis developed in *Lmna*^H222P/H222P^ mouse model also sheds light on the role of cardiac fibroblast (Arimura et al., 2005; Raman et al., 2007; Wu et al., 2011), a process, less understood. Our results imply a potential pathogenic role for cardiac fibroblasts in *LMNA*-DCM beyond just myocyte defects. Our results further validate the iPSC model for *LMNA*-associated disease by the recapitulation of some of the fundamental defects of the disease as of humans. Notably, gene expression, signal transduction, cellular structure, and functional aberrations of iCMs and iCFs bearing different *LMNA* mutations are not uniform. These differences may, in part, be due to the specific mutation sites within the *LMNA* gene. Detailed characterizations and comparisons are summarized in Table 2 and Table 3.

**Table 2:** Phenotype comparison of iCMs carrying different *LMNA* mutations.

Subject	Lamin A/C protein	pERK/total ERK	Cleaved caspase -3	Nuclear cir- cularity	Sar- comere dis array	mRNA							Cx43 pro- tein	Ca^2+^ transient				MEA			
						*SCN5A*	*KCNH2*	*KCNQ1*	*ATP 2A2*	*PLN*	*CASQ2*	*GJA1*		Alter nans	Brady cardia	EAD	Oscil/ Fib	Beat period	FPDc	CV	E-C delay
M1I	↓↓	↔	↔	↔	↑	↓	↓	↓	↑↑	↑	↑↑	↔	↔	↑	↔	↑	↑	↑	↑	↓	↑
R216C /R399H	↓	↔	↑	↔	↑	↔	↔	↔	↔	↔	↔	↔	↔	↑	↔	↑	↑	↑	↑	↓	↑
R216 C.m	↔	↔	↑	↔	↑	↔	↔	↔	↔	↔	↔	↔	↔	↔	↔	↑	↑	↔	↑	↔	↔
R216C.f	↓	↑	↑	↔	↑	↓	↔	↓	↑	↑	↔	↑	↔	↔	↔	↑	↑	↑	↑	↔	↔
R335Q	↓	↑↑	↔	↔	↑	↓	↔	↓	↔	↔	↔	↓	↔	↔	↑↑	↑	↑	↑	↑	↓	↑
R377H	↓	↑	↑	↔	↑	↓	↔	↔	↑↑	↑	↑↑	↓	↔	↔	↑↑	↑	↑	↑	↑	↓	↑
R541C	↓	↑↑	↔	↔	↑	↓	↓	↓	↔	↔	↔	↔	↔	↔	↑↑	↑	↑	↑	↑	↓	↑

Symbol ↑ indicates an increase; ↓ decrease; ↔ no significant change; MEA indicates micro electrode array; EAD early afterdepolarization; Oscil/Fib oscillations/fibrillations; FPDc rate-corrected field potential duration; CV conduction velocity; E-C delay excitation contraction delay.

**Table 3:** Phenotype comparison of iCFs carrying different *LMNA* mutations.

Subject	Lamin A/C protein	pERK/total ERK	Cleaved caspase 3	*GJA1* transcript	Cx43 protein	Nuclear circularity	Migration rate
M1I	↓	↑↑	↔	↓	↓↓	↔	↓
R216C/R399H	↔	↑	↔	↓	↓	↓	↓
R216C.m	↔	↑	↔	↓	↓	↓	↓
R216C.f	↔	↑	↔	↓	↓	↓	↓
R335Q	↔	↔	↔	↓	↓	↓↓	↓↓
R377H	↑↑	↑↑	↔	↓	↓	↓↓	↓
R541C	↑↑	↑↑	↔	↓	↓↓	↔	↓

Symbol ↑ indicates an increase; ↓ decrease; ↔ no significant change.

The lamins couple the nucleus to the cytoskeleton by anchoring the linker of nucleoskeleton and cytoskeleton complex and thus, controls nuclear position, shape, and mechanochemical signaling (Chang et al., 2015; Brayson and Shanahan, 2017). Previously, Chatzifrangkeskou et al. reported a disrupted sarcomere organization in CMs carrying the *LMNA* p.R190W mutation (Chatzifrangkeskou et al., 2018). Similarly, our seven *LMNA* mutant iCM lines showed that the sarcomeres were highly disorganized. Immunofluorescence imaging of lamin A/C in iCMs failed to demonstrate obvious nuclear morphological deformities that have been observed in iCMs with other *LMNA* mutations (Siu et al., 2012; Shah et al., 2019). In those studies however, nuclear deformities were only obvious after prolonged stress was applied by either electrical stimulation or ischemia. Higher resolution imaging with electron microscopy may review more subtle nuclear membrane abnormalities. Either insufficient lamin A/C abundance or the presence of malfunctioning mutant lamin A/C proteins may impair the structural integrity of the nuclear lamina. This instability may alter nuclear mechanotransduction processes with dysregulation of ensuing downstream processes such as mechanical/electrical activity, epigenetic changes in chromatin, gene expression, DNA damage, and cell death (Chen et al., 2019; Donnaloja et al., 2020). The latter is also supported by increased caspase-3 activity that we observed in iCMs and is consistent with results from a previous study of *LMNA* p.S143P iCMs (Shah et al., 2019). In contrast to the *LMNA*-iCM lines, nuclear deformity was markedly more pronounced in the corresponding *LMNA*-iCF lines. The *LMNA* mutations in our iCFs likely lead to lamina stability with greater susceptibility in iCFs to nuclear envelope deformity nuclei of varying degrees. *LMNA* mutation-mediated cytoskeleton disruption may inhibit its actin-bundling and interrupt nuclear positioning (Antoku et al., 2019), resulting in impaired fibroblast migration, consistent with the varying impairments we observed in the wound healing assay. It is interesting that lamin A/C abundance in the iCMs was generally reduced compared with controls while in iCFs two lines exhibited increased expression, including R541C which came from a family with markedly early-onset and aggressive DCM. This would suggest that the relatively high expression of mutant protein may correlate with the clinical severity involving cardiac fibroblast abnormality.

A homozygous mutant *Lmna*^H222P/H222P^ mouse model based on a human mutation associated with Emery-Dreifuss muscular dystrophy has been the basis for much of the molecular mechanistic studies of *LMNA*-DCM (Muchir et al., 2007; Muchir et al., 2009). This mouse developed DCM preceded by activation of MAPK pathways involving TGF-b/Smad signaling and connective tissue growth factor (Chatzifrangkeskou et al., 2016). Based on data from this model, inhibition of the p38a pathway has reached Phase III clinical trials (Pfizer ARRY-371797). Other inhibitors (MEK1/2 and JNK) converging on MAPK and AKT/mTOR pathways, as well as NAD+ supplementation, have been promoted as therapeutic approaches using this model (Choi and Worman, 2013; Vignier et al., 2018). These results point to an encouraging future of precision medicine in *LMNA*-DCM however, there is a limitation to solely relying on the *Lmna*^H222P/H22P^ model. CLINVAR reports 1,250 variants in *LMNA*. Of these, 387 are categorized as Pathogenic or Likely Pathogenic (a conservative estimate). To assume *L*-H222P pathogenic mechanisms can be generalized to all of these is premature. Our results demonstrate variability in the hyperactivation of pERK across patient-specific iCMs and iCFs with some showing no difference from control lines. Such an approach utilizing patient- or mutation-specific iPSCs may serve as a predictor of who will respond to new therapies targeting gene-associated diseases.

The abnormal electrophysiology characteristics we observed among the different *LMNA*-iCMs further support phenotypic diversity among differing genotypes. The variation in ion channel expression deviations among *LMNA*-iCMs may underly the diversity in phenotypes. Altered expression of sodium channels and connexins have been implicated in arrhythmias that are prevalent in *LMNA*-DCM and other DCMs. In line with previous discoveries on *LMNA*-N195K (Mounkes et al., 2005), S143P (Shah et al., 2019) and K219T (Salvarani et al., 2019), our MEA recordings revealed a reduced beating rate and conduction velocity as well as increased FPD and excitation-contraction coupling combined with a higher frequency of arrhythmias on calcium imaging as a result of diverse *LMNA* mutations. The detailed mechanisms of increase in beating rhythm variability at the cellular level remain incompletely understood, but our results suggest *LMNA*-associated alterations of ion channel and calcium signaling gene expression as a factor. Interestingly, no significant difference in Cx43 protein levels was observed in iCMs *via* immunoblot (despite significant changes in transcription) whereas all *LMNA*-iCF lines had varying degrees of reduced Cx43 protein. To determine if *LMNA*-iCFs could participate in the pathophysiology of *LMNA*-DCM, we cocultured iCFs with iCMs from the same iPSC parent cell line. The addition of 10% of iCFs in the iCM culture showed a trend towards enhanced *LMNA*-associated electrophysiological abnormalities assayed on the MEA system compared to iCMs alone, suggesting that cardiac fibroblasts interact with, and adversely affect cardiomyocyte function in *LMNA*-associated DCM.

Each type of disease model system has its advantages and disadvantages and offers complementary insights into disease mechanisms and treatment approaches. Our results highlight several benefits of the iPSC approach including patient-specificity, time, cost, and species relevance (Milani-Nejad and Janssen, 2014). Furthermore, the ability to dissect cell-type specificity contributing to pathogenesis is a promising avenue that may enhance therapeutic target development. It is important to acknowledge the limitations of this approach also. Even though iCMs are reported to closely resemble adult human cardiomyocytes, they still have less mature phenotypes (Karbassi et al., 2020). Variations among the *LMNA* cell lines may be due to different mutation sites however, the genetic background may also influence phenotypic expression. This is apparent when we examine phenotypic differences between the R216C.m and R216C.f lines harboring identical *LMNA* mutations but from different families and of different gender (Figures 2A,B, 5C, 7A,C,E,G). Accordingly, isogenic control lines are required to fully explore the effects of a given genetic mutation. We are embarking on gene editing of these cell lines using CRISPR-Cas9 approaches (Anzalone et al., 2019). Nevertheless, our study illustrates the differences and similarities of the phenotypic hallmarks associated with various *LMNA* mutations. Further investigation is merited to identify detailed mechanisms focusing on the fibrosis pathway, the direct/indirect interplay between iCMs and iCFs, and the role of endothelial cells and macrophages derived from iPSCs in the development of *LMNA* related cardiomyopathy. Potential therapeutic targets (molecular- and cell-type-specific) that are candidates for further exploration for early- or pre-clinical stages of *LMNA* mutation carriers may be identifiable with this approach.

## Data Availability Statement

The original contributions presented in the study are included in the article/Supplementary Material, further inquiries can be directed to the corresponding author.

## Ethics Statement

The studies involving human participants were reviewed and approved by the Institutional Review Board of the University of South Florida (IRB: Pro00033948). The patients/participants provided their written informed consent to participate in this study.

## Author Contributions

TM and JY: project conception and design. JY, MAA, MBA, AB, and MB: acquisition of data. JY and TM: analysis and interpretation of data. JY, MAA, MBA, AB, MB, and TM: writing and editing of manuscript. All authors contributed to the article and approved the submitted version.

## Funding

This work was supported by USF-Preeminence Fund and MAA was supported by The W.Paul Hoenle Foundation.

## Conflict of Interest

The authors declare that the research was conducted in the absence of any commercial or financial relationships that could be construed as a potential conflict of interest.

## Publisher’s Note

All claims expressed in this article are solely those of the authors and do not necessarily represent those of their affiliated organizations, or those of the publisher, the editors and the reviewers. Any product that may be evaluated in this article, or claim that may be made by its manufacturer, is not guaranteed or endorsed by the publisher.
