# Corrigendum: Phenotypic variability in iPSC-induced cardiomyocytes and cardiac fibroblasts carrying diverse *LMNA* mutations

**DOI:** 10.3389/fphys.2022.974151

**Published:** 2022-08-31

**Authors:** Jiajia Yang, Mariana A. Argenziano, Mariana Burgos Angulo, Alexander Bertalovitz, Maliheh Najari Beidokhti, Thomas V. McDonald

**Affiliations:** ^1^ Department of Molecular Pharmacology and Physiology, Morsani College of Medicine, University of South Florida, Tampa, FL, United States; ^2^ Heart Institute, Department of Medicine (Division of Cardiovascular Sciences), Morsani College of Medicine, University of South Florida, Tampa, FL, United States

**Keywords:** *LMNA*, dilated cardiomyopathy, induced pluripotent stem cell, cardiomyocytes, cardiac fibroblasts, connexin 43

In the published article, there was an error in Figure 1 as published. In panel B, ALP staining pictures for R335Q and R377H were inadvertently mislabeled, which needs to be replaced. R335Q shows overlap with M1I, and R377H shows overlap with R541C. The corrected Figure 1 appears below.

**FIGURE 1:**
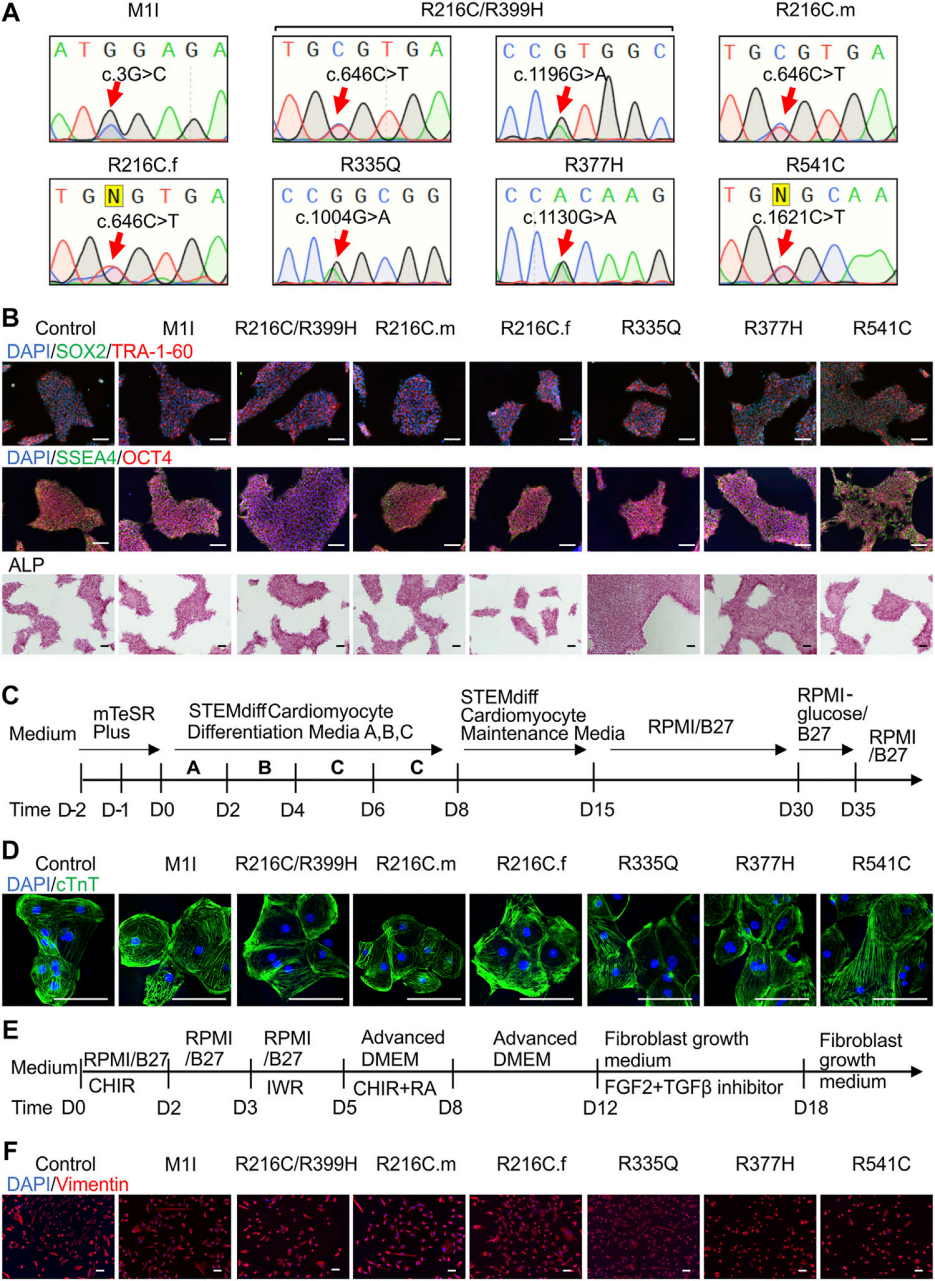
Differentiation and characterization of induced pluripotent stem cell (iPSC)-derived cardiomyocytes (iCMs) and cardiac fibroblasts (iCFs). **(A)** Sanger sequencing validates the presence of the individual mutation. **(B)** Representative staining of iPSCs expressing pluripotency markers SOX2 (green) and TRA-1-60 (red), SSEA4 (green), OCT4 (red), and alkaline phosphatase. Nuclei were stained with DAPI (blue). **(C)** Schematic of cardiac differentiation using STEMdiff™ Cardiomyocyte Differentiation kit. **(D)** Immunostaining of cardiac troponin T positive cardiomyocytes. **(E)** Workflow to induce cardiac fibroblasts using small molecule-based protocols. **(F)** Immunostaining of cardiac fibroblast specific marker, vimentin. Scale bar, 100 μm.

The authors apologize for this error and state that this does not change the scientific conclusions of the article in any way. The original article has been updated.

