# Supplementary material for: Phenotypic Variability in iPSC-Induced Cardiomyocytes and Cardiac Fibroblasts Carrying Diverse LMNA Mutations
Source: Front Physiol. 2021 Dec 16;12:778982. doi: 10.3389/fphys.2021.778982 (PMC8716763; doi:10.3389/fphys.2021.778982)
Supplement: Supplementary file 1 [file Data_Sheet_1.PDF]

SUPPLEMENTAL MATERIAL

Table S1. Antibodies used for immunofluorescence/immunoblotting.

	Antibody	Application	Dilution	Company Cat #
Primary	Mouse anti-SSEA4	Immunofluorescence	1:200	Thermo Fisher Scientific Cat# 41-4000
	Rabbit anti-OCT4		1:200	Thermo Fisher Scientific Cat# PA1-16943
	Rabbit anti-SOX2		1:200	Thermo Fisher Scientific Cat# PA1-094
	Mouse anti-TRA-1-60		1:200	Thermo Fisher Scientific Cat# 14-8863-82
	Mouse anti-cTnT		1:200	Santa Cruz Biotechnology Cat# sc-20025
	Rabbit anti-vimentin		1:200	Abcam Cat# ab 45939
	Mouse anti-lamin A/C		1:100	Thermo Fisher Scientific Cat# MA3-1000
	Mouse anti-α-actinin		1:800	Sigma-Aldrich Cat# A7811
	Rabbit anti-connexin 43		1:200	Cell Signaling Technology Cat# 3512s
		Immunoblot	1:1000	
	Rabbit anti-pERK		1:1000	Cell Signaling Technology Cat# 9101s
	Mouse anti-total ERK		1:1000	Santa Cruz Biotechnology Cat# sc-514302
	Rabbit anti-cleaved caspase 3		1:1000	Cell Signaling Technology Cat# 9661s
	Mouse anti-lamin A/C		1:1000	Santa Cruz Biotechnology Cat# sc-376248
	Rabbit anti-PAX6	Immunofluorescence	1:200	Thermo Fisher Scientific Cat# 42-6600
	Mouse anti-Nestin		1:200	Thermo Fisher Scientific Cat# MA1-110
	Goat anti-brachyury		1:50	R&D system AF2085
	Mouse anti-α-SMA		1:200	Abcam ab7817
	Rabbit anti-SOX17		1:200	Proteintech Cat# 24903-1-AP
	Mouse anti-AFP		1:200	Thermo Fisher Scientific Cat# MA1-19178
Secondary	Donkey Anti-Mouse IgG (H+L) Antibody, Alexa Fluor 488 rabbit		1:500	Thermo Fisher Scientific Cat# A-21202
	Donkey anti-Mouse IgG (H+L) Antibody, Alexa Fluor 594		1:500	Thermo Fisher Scientific Cat# A-21203
	Donkey anti-Rabbit IgG (H+L) Antibody, Alexa Fluor 488		1:500	Thermo Fisher Scientific Cat# A-21206
	Donkey anti-Rabbit IgG (H+L) Antibody, Alexa Fluor 594		1:500	Thermo Fisher Scientific Cat# A-21207
	Donkey anti-goat IgG (H+L) Antibody, Alexa Fluor 647		1:500	Thermo Fisher Scientific Cat# A-21447
	IRDye® 800CW Donkey anti-Mouse IgG	Immunoblot	1:10000	LI-COR Cat# 925-32212
	IRDye® 800CW Donkey anti-Rabbit IgG		1:10000	LI-COR Cat# 925-32213
	IRDye® 680RD Donkey anti-Mouse IgG		1:10000	LI-COR Cat# 925-68072
	IRDye® 680RD Donkey anti-Rabbit IgG		1:10000	LI-COR Cat# 925-68073

Table S2. Primer sequences used for PCR.

	**Target**	**Size of band**	**Forward/Reverse primer (5′-3′)**
Pluripotency Markers (PCR)	Nanog	189 bp	F: CCATCATTTCCGAGTGCAAGTGCT<br>R: AAGCTAGGTCTCTGTAGCCCAGAA
Pluripotency Markers (PCR)	c-Myc	328 bp	F: GCGTCCTGGGAAGGGAGATCCGGAGC<br>R: TTGAGGGGCATCGTCGCGGGAGGCTG
Pluripotency Markers (PCR)	Oct4	145 bp	F: GACAGGGGGAGGGGAGGAGCTAGG<br>R: CTTCCCCTCCAACCAGTTGCCCCAAAC
Pluripotency Markers (PCR)	Sox2	151 bp	F: GGGAAATGGGAGGGGTGCAAAAGAGG<br>R: TTGCGTGAGTGTGGATGGGATTGGTG
Pluripotency Markers (PCR)	Klf4	397 bp	F: TGATTGTAGTGCTTTCTGGCTGGGCTCC<br>R: ACGATCGTGGCCCCGGAAAAGGACC
Genotyping	*LMNA*	715bp	F: CTCAGCCAGCTCCAGAAG<br>R: TCATCCTCGTCGTCCTCAA

Supplemental Figure 1

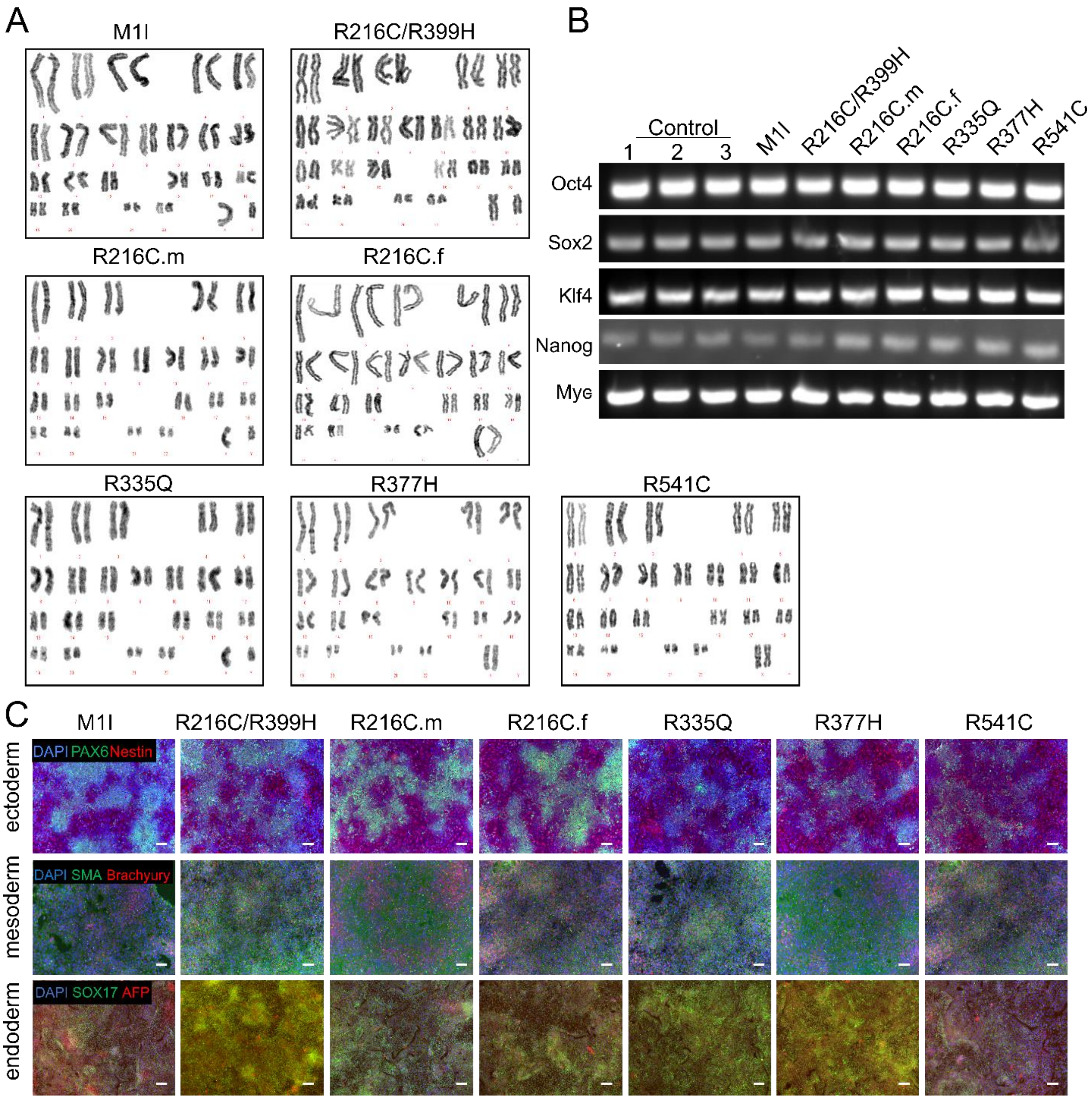

Supplemental Figure 1. Characterization of iPSCs.

(A) G-banding karyotype image demonstrating normal karyotype of reprogrammed iPSCs. **(B)** PCR analysis showing the presence of pluripotency markers in iPSCs. (**C**) Immunofluorescence micrographs demonstrating trilineage differentiation into ectoderm (PAX6, Nestin), mesoderm (α-SMA, Brachyury) and endoderm (SOX17, AFP). Scale bar, 100 µm.
